# Ion Channel Targeting with Antibodies and Antibody Fragments for Cancer Diagnosis

**DOI:** 10.3390/antib8020033

**Published:** 2019-05-24

**Authors:** Claudia Duranti, Annarosa Arcangeli

**Affiliations:** Department of Experimental and Clinical Medicine, Section of Internal Medicine, University of Florence, 50134 Firenze, Italy; claudia.duranti@unifi.it

**Keywords:** antibodies, cancer diagnostics, in vivo imaging, engineered antibody fragments, hERG1

## Abstract

The antibody era has greatly impacted cancer management in recent decades. Indeed, antibodies are currently applied for both cancer diagnosis and therapy. For example, monoclonal antibodies are the main constituents of several in vitro diagnostics, which are applied at many levels of cancer diagnosis. Moreover, the great improvement provided by in vivo imaging, especially for early-stage cancer diagnosis, has traced the path for the development of a complete new class of antibodies, i.e., engineered antibody fragments. The latter embody the optimal characteristics (e.g., low renal retention, rapid clearance, and small size) which make them ideal for in vivo applications. Furthermore, the present review focuses on reviewing the main applications of antibodies and antibody fragments for solid cancer diagnosis, both in vitro and in vivo. Furthermore, we review the scientific evidence showing that ion channels represent an almost unexplored class of ideal targets for both in vitro and in vivo diagnostic purposes. In particular, we review the applications, in solid cancers, of monoclonal antibodies and engineered antibody fragments targeting the voltage-dependent ion channel Kv 11.1, also known as hERG1.

## 1. Introduction

Antibodies have become common and essential research instruments over the last fifty years, providing highly specific and versatile tools for a wide array of experimental applications in many fields. Furthermore, monoclonal antibodies (mAbs) and, more recently, recombinant antibodies have gained clinical applications for diagnosis and therapy of different diseases, including cancer [1,2]. The translation of antibodies from basic research into the clinic has therefore significantly changed the prognosis for different classes of human cancers. The great success of the clinical application of antibodies mainly relies on the high versatility of these biological molecules. Indeed, antibodies combine the specificity of antigen targeting with, e.g., the possibility to be easily conjugated with various molecular or chemical agents, thus improving their pharmacological efficacy. Targeted therapeutic strategies have recently raised the possibility of tailoring cancer treatment to an individual patient after assessing the peculiar molecular characteristics of the tumor under treatment. Such refined diagnostic assessments can be achieved using antibody molecules. The fusion between diagnostic imaging techniques and therapeutic intervention is important to address cancer heterogeneity [3]. The term “*theranostics”* was hence coined to describe a molecular tool having both diagnostic and therapeutic applications [4]. Moreover, several platforms linking a diagnostic tool, often represented by an antibody, with a defined therapeutic compound have been developed and marketed. Such “companion diagnostics” are embodying an indispensable part of personalized cancer medicine [5].

The present review focuses on reviewing the main applications of mAbs for cancer diagnosis in vitro. Moreover, we address how the technology of engineering antibody molecules, and in particular the possibility of developing antibody fragments, is greatly impacting on in vivo molecular imaging, for diagnostic applications in solid cancers. We also provide strong evidence that ion channels are relevant molecular devices in cancer establishment and progression, and that can be exploited for either in vitro or in vivo cancer diagnosis. In particular, the diagnostic and prognostic applications, in solid cancers, of mAbs and antibody fragments targeting the voltage-dependent ion channel K_v_11.1, also known as hERG1, are thoroughly discussed.

## 2. Antibody-Based Cancer Diagnostics

Solid cancer diagnosis is currently based on imaging techniques (e.g., Computer-Assisted Tomography, Magnetic Resonance Imaging, etc.), laboratory assays (e.g., tests for circulating tumor markers such as the carcinoembryonic antigen) and the pathological evaluation of either biopsies or surgical specimens. The latter can take advantage of either biomolecular techniques or antibody-based immunohistochemistry (IHC) to provide further insights for patients’ prognostic stratification and therapeutic choice. The number and type of techniques available to allow physicians to detect and diagnose cancer had significant changes in the last years. In fact, more accurate and reproducible imaging techniques have been developed and applied to the clinical setting. Moreover, novel cancer biomarkers have been identified to improve diagnosis and prognosis. In this scenario, antibodies represent key devices for both in vitro and in vivo diagnosis, since they can specifically recognize specific cancer biomarkers in tissues and body fluids. In particular, while mAbs represent good molecular tools to detect cancer biomarkers in vitro, in tissue specimens, their use in vivo is hindered by several concerns (see Section 3.2) and are progressively being substituted by antibody fragments [6].

Hereafter, the main antibody-based in vitro and in vivo techniques for cancer diagnosis are reported.

### 2.1. In Vitro Cancer Diagnostics

Solid cancer diagnosis in vitro is now routinely improved by the detection of clinically validated biomarkers through IHC on paraffin-embedded tissue slides. After antibody binding to the specific antigen, the target region can be visualized by an enzyme-linked (e.g., horseradish peroxidase) or a fluorescent dye, a radioactive tracer or a colloidal gold reagent. The positivity of the tumor for a given marker is hence evaluated, applying predetermined cutoffs. New IHC techniques have improved both the optical resolution and the sensitivity of detection, mainly through the use of amplification procedures, despite the risks of false-positive and false-negative staining [6]. Some “in vitro diagnostics” (IVD) based on antibodies (and the related IHC technique) have been clinically validated and are currently applied in the clinical practice (see Table 1). mAbs can also be utilized as “companion diagnostics”, i.e., diagnostics that can be associated with the use of a particular treatment, either a small molecule or a therapeutic antibody. The path to companion diagnostics started in 1998 with the approval of the therapeutic humanized mAb Trastuzumab, which was paralleled by the simultaneous approval of a diagnostic test, the HercepTest. Some of the approved companion diagnostics are reported in Table 1.

An emerging application of antibodies for cancer diagnostic and prognostic purposes relies on the use of “omics” data [7]. However, both IHC and omics strategies display some disadvantages, since they can hardly address tumor heterogeneity. Furthermore, tumor biopsies are not always able to reveal the overall antibody binding or the overall expression of the biomarker. Both hindrances have been recently addressed through the so called “liquid biopsy”, aimed at the detection of either circulating, cell-free, DNA or of circulating tumor cells (CTCs). CTCs detection can help to monitor a patient’s condition, including the tracking of the genomic evolution of the tumor. CTC studies are accomplished through microfluidics platforms, and a new approach includes the use of EpCAM expression levels for the capture of CTCs. Besides anti-EpCAM antibodies, other antibodies targeting specific tumor biomarkers are now under development to improve microfluidic-based CTC analyses [8].

### 2.2. In Vivo Cancer Diagnostics: Molecular Imaging

The accuracy that allows antibodies to precisely identifying their targets has stimulated their application also for in vivo imaging, thus improving the diagnostic imaging approaches currently used. For example, the 18F-Fluoridexyglucose-Positron Emission Tomography (FDG-PET) imaging represents an invaluable diagnostic and prognostic tool to detect high levels of glycolytic activity, a sign of malignant transformation. However, FDG-PET is not free of false negatives (e.g., tumors with more indolent growth or dependent on different metabolic pathways) or false positives, as in the case of infection or inflammation [9]. Notably, the majority of imaging probes actually in use to detect cancer can also detect inflammation, thus leading to significant numbers of potential false positives. Hence, using antibodies for in vivo imaging may lead to a versatile approach which allows more accurate diagnosis, staging and hence disease management. Some practical examples of mAbs recognizing cancer specific biomarkers that are approved by the FDA and/or EMA and are currently used in the clinical setting are shown in Table 2A. Among these, ProstaScint is a mAb used in prostate cancer patients as a diagnostic imaging agent to detect nodal metastases “pre-prostatectomy”, or recurrence in post prostatectomy patients with a rising prostate-specific antigen (PSA). The therapeutic mAbs, cetuximab and trastuzumab, are also used as in vivo tracers. In particular, the dual-labeled (^111^In-DTPA)n-trastuzumab-(IRDye800)m is capable of tracking HER2 overexpression in breast cancer patients [10]. cetuximab has been repurposed for fluorescent imaging and is in phase I and phase II clinical trials for malignant glioma and pancreatic cancer imaging and fluorescence-guided surgery with IRDye-800CW [11].

Although antibodies have definitely improved cancer diagnostics through their application in IVD kits, the use of mAbs as molecular imaging tools for in vivo diagnostics still needs further improvements. In other words, antibodies need to be designed differently for their use as diagnostics in vitro and for their application as in vivo imaging agents. When injected in vivo, in fact, whole antibodies display long serum half-lives (1–3 weeks), which are favorable if they are envisaged to be applied as therapeutics, thanks to the enhancement of the exposure of target tissues to the antibody. Moreover, the effector domain (crystallizable fragment, Fc) often exerts biological activities which are essential for the therapeutic functions of the whole antibody molecule [12]. However, such characteristics are drawbacks for the use of such molecules as imaging agents, as several days are required to obtain a good signal-to-noise ratio, and a biological activity is undesirable for an imaging agent.

Many of the disadvantages of whole antibody molecules have been overcome thanks to antibody engineering, producing smaller and highly versatile molecules [13], which maintain the specificity of mAbs but allow higher tumor penetration and shorter clearance times, both optimal characteristics for diagnostic purposes.

## 3. Antibody Fragments for Cancer Diagnostics

**Figure 1:**
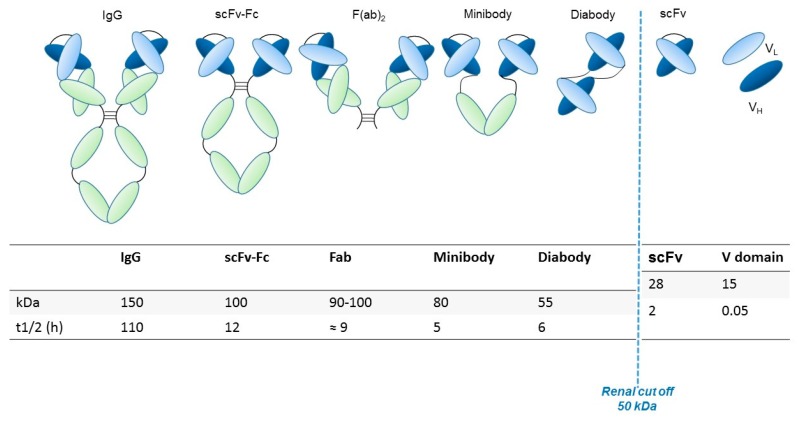
Engineered antibody formats. Different antibody formats are reported in the figure, showing their different size compared to intact IgG. Ig, Immunoglobulin; scFv, single-chain variable fragment; V domain, variable domain. In the figure, the size (KDa) of each different antibody format and their half-lives (t_1/2_, i.e., time needed to eliminate half of the molecule from circulation) are also reported.

Generally, the preservation of the variable fragment (Fv) domain in engineered antibody fragments preserves the antigen binding. Conversely, the absence of the Fc region abolishes the immune interactions mediated by the complement and by other effectors. The elimination of the Fc region also blocks recycling through the path of the neonatal Fc receptor (FcRn), facilitating the contrast and thus the visualization of targeted tissues through rapid clearance from the blood [13]. Smaller fragments allow the use of radionuclides that decay rapidly (for example through labeling with 18F), thus resulting in reduced radiation exposure. Furthermore, the use of antibodies with a reduced molecular weight (below ~60 kDa) speeds up the elimination through the renal clearance [14]. The main structural characteristics, molecular weight and renal clearance of different engineered antibody fragments are shown in Figure 1 and described in the following paragraph.

### 3.1. Antibody Fragments: Characteristics and Development

A comparison of the pharmacokinetics and target specificities between intact and fragmented antibodies has been performed, and the results show that recombinant antibodies retain the antigen specificity of the intact Igs from which they derive advantageous characteristics regarding tumor penetrance and retention. Furthermore, antibody fragments have several formats which are reported in Figure 1: scFv-Fc (≈ 100 KDa), minibodies (Mb; scFv-CH3 dimers, ≈ 80 kDa), F(ab’)2 and Fab (≈ 50 KDa), bispecific antibodies (≈ 55-60 KDa) and scFv (≈ 25 KDa) [12]. The F(ab) fragment is an antibody structure that still binds to antigens but is monovalent and lacks the Fc portion. An antibody digested by the enzyme papain yields two F(ab) fragments of about 50 kDa each and an Fc fragment [15].

Another class of antibody fragments is based on single-domain antibodies. 

Among sdAbs, the so called nanobodies merit attention. Nanobodies (~15kDa) are fragments of heavy single-variable heavy chains (VH_H_s), originated from antibodies found in the camelids. The small size of these antibodies allows them to bind to epitopes to which intact molecules cannot access [16] and makes them appropriate for applications where an extremely short time of clearance is desired. The latter is also determined by the size, the charge and by the presence/absence of conjugated parts. Fragments with weights lower than the renal threshold (~60 kDa) are eliminated through the kidneys, while larger molecules are instead cleared through the liver. Nanobodies can be used in diagnostics both at the initial stages for the detection of the tumor itself and then to evaluate the expression of a target for therapeutic intervention or for monitoring of the disease. This class of molecules can be coupled with various nanocarriers (e.g., iron oxide nanoparticles, silica nanoparticles, gold nanostructures, and carbon nanomaterials), which could be suitable for anticancer drug delivery [17].

From the diagnostic point of view, two important classes of antibody fragments are the scFvs, single-chain variable fragments, and the bispecific antibodies. scFvs have a molecular weight around 25 kDa and are composed of V_H_ and V_L_ chains, joined via a flexible peptide linker. The first scFv molecules were developed in 1988 and represent the smallest functional V_H_–V_L_ domains of an antibody necessary for the high-affinity binding of an antigen. Peptide linkers are fundamental for the assembly of functional scFv antibodies, as they join the VH and VL chains and usually vary from 10 to 25 amino acids in length and typically include hydrophilic amino acids. The most common linker is the decapenta-peptide (Gly4Ser)3. The variable regions can be connected in either the VH-linker-VL (most common) or VL-linker-VH orientation. In any case, such orientations can affect expression efficiency, stability and antigen binding activity [18].

Another class of antibodies which has gained great attention is that of bispecific antibodies, among which single-domain diabody (scDb) is the most versatile format. The first work on bispecific antibody generation was published in 1961 and described the production of chimeric antibodies containing two different antigen-binding sites simultaneously. Such molecules are capable of binding two different antigens at the same time, thus allowing the recognition of two different targets which might be crucial in the diagnostic setting. scDbs conjugate the bispecificity with the characteristics (low molecular weight, high tissue penetration, and good clearance times) of antibody fragments [19].

### 3.2. Applications of Antibody Fragments for in Vivo Imaging

Recent examples of antibody fragments used as in vivo diagnostics combined encompass several of the aforementioned formats even though this field offers great space for improvement as few of such molecules are actually used for in vivo imaging diagnostic applications. Among those few are many Fab fragments for HER2 (human epidermal growth factor receptor 2) targeting and the use of ^124^I-PSCA (Prostate Stem Cell Antigen)-specific minibody in order to assess the response to prostate cancer treatment using enzalutamide [9]. Moreover, F(ab’)2 and Fab fragments radiolabeled with ^111^In (Indio111) and minibodies and diabodies labeled with 89Zr (Zirconium 89) have been used for imaging with SPECT (Single Photon Emission Computed Tomography) or PET (Positron Emission Tomography) in small animals targeting the prostate-specific membrane antigen (PSMA). The great interest in fragments of antibodies is demonstrated by several papers using nanobodies [20,21] and affibodies [22]. So far, preclinical studies have been performed to give insights into the biochemical and biophysical features of several antibody fragments as imaging agents [23,24]. Ogasawara and colleagues demonstrated that antibodies specific for phosphatidylserine can be a valuable tool to assess cell death in response to treatment [25]. Direct imaging with antibodies could also offer a suitable technique to determine the development of resistance to therapy. Li and colleagues [9] focused on MET receptor, using various antibody fragments derived from an anti-MET antibody to obtain images of non-small-cell lung cancer xenografts from cell lines resistant to targeted anti-EGFR therapy due to the overexpression of MET.

Finally, bispecific radioimmunoconjugates (bsRICs) capable of binding to HER2 and EGFR were developed for both therapy and diagnostic applications and tested in preclinical models. The aim of these studies was to better decipher the mechanisms underlying trastuzumab resistance related to the overexpression of EGFR in patients with HER2-positive carcinomas. These bsRICs are composed of the trastuzumab Fab (ligand for HER2) linked to EGF (ligand for EGFR) via a long spacer (polyethylene glycol). Such antibodies show good specificity and low uptake in normal organs except for the kidneys [26]. We report, in Table 2, the main monoclonal and engineered antibody fragments which are used in vivo and have already been approved by the FDA and/or EMA.

## 4. Ion Channels in Cancer

Ion channels are membrane proteins which, besides controlling cell excitability and ionic and fluid homeostasis, are emerging to be particularly relevant in cancer [27]. In particular, being mainly present on the plasma membrane of cancer cells, ion channels can mediate the cross-talk between tumor cells and the tumor microenvironment to drive different features of neoplastic progression (e.g., cell proliferation and survival, cell invasiveness, and pro-angiogenic programs) [28]. What is more, ion channels represent one of the rare druggable molecular classes and are increasingly recognized as novel and valuable molecular targets for antineoplastic therapy [29]. Some ion channel modulators, previously used in not oncological settings, are currently in clinical trials for cancer treatment (https://clinicaltrials.gov/).

Ion channels are involved in tumor progression through different mechanisms. For example, K^+^ channels allow uncontrolled tumor cell proliferation by setting the membrane potential (Vm) to rather depolarized values. The Ca^2+^-dependent K^+^ (K_Ca_) channels can couple V_m_ to variations of the intracellular Ca^2+^ concentration ([Ca^2+^]_i_). The latter is a finely tuned process that involves both plasma membrane (e.g., ORAI1 and TRPC1 channels) and intracellular (e.g., STIM1) proteins. In several types of cancer cells, [Ca^2+^]_i_ regulates critical cellular processes such as gene expression and motility. Another channel type likely relevant to cancer is the volume-regulated anion channel VRAC, formed by a multimeric assembly of LRRC8A–LRRC8E proteins. The regulatory effects of VRAC on cellular volume (in particular in the process called apoptotic volume decrease (AVD)) play a crucial role in cancer progression and metastasis, as well as in drug resistance.

**Figure 2:**
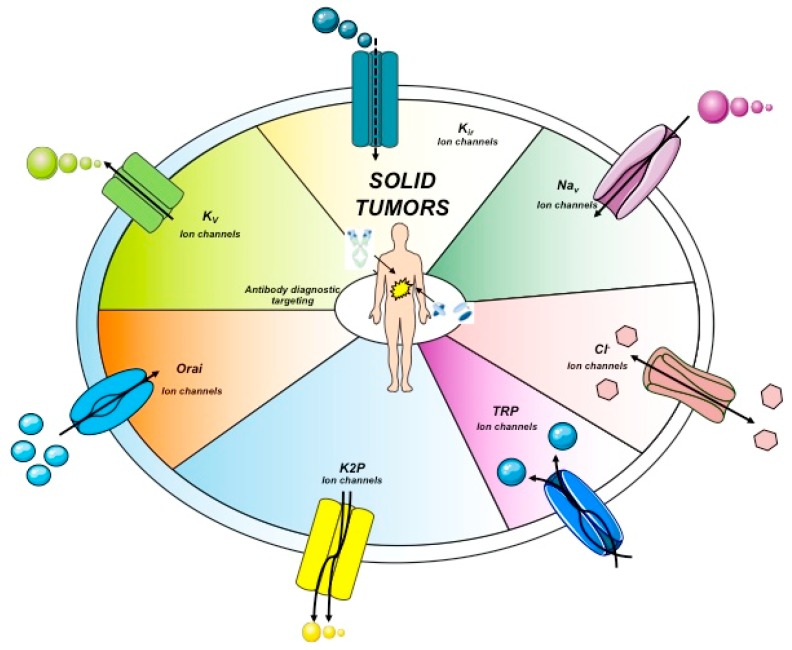
Ion channels topology expressed in solid cancers. The main ion channels and their structures are reported. These represent the main proteins expressed in solid cancers. K_ir_, inward-rectifier potassium channel.

**Figure 3:**
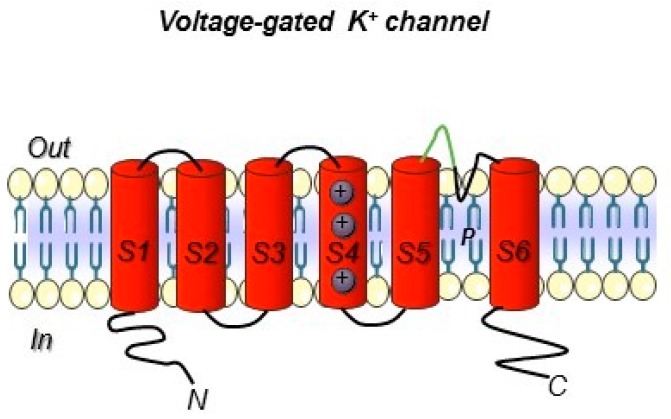
Voltage-gated K^+^ ion channel topology. The six transmembrane segments S1–S6 are reported. The S5 and S6 domain are connected with a loop that control selectivity. In green, the loop portion towards which the hERG1-mAb was developed is highlighted (see the following paragraphs). Both the N- and C- terminus are intracellular.

Another anion channel frequently upregulated in cancer is the Ca^2+^-activated Cl^-^ channel TMEM16A, also called ANO1. Voltage-gated sodium (Na_v_) channels are often expressed de novo in carcinomas. Besides contributing to tumor “electrical excitability”, they can regulate intracellular Na^+^ concentrations [Na]_i_ and in turn activate Na^+^-driven exchangers. In any case, Na_v_ are mainly involved in the triggering of cell invasiveness and metastatic spread. The Transient Receptor Potential (TRP) channels are also dysregulated in cancer, where they can also operate in conjunction with growth factor receptors: TRPC1 binds to the Fibroblast Growth Factor Receptor (FGFR1) and drives cell proliferation by a modulation of bFGF-triggered Ca^2+^ signals; the TRP Ankyrin 1 (TRPA1) channel binds to FGFR2 in lung adenocarcinoma cells, and activates the metastatic process thanks to this strict binding. Some cancer-related ion channels operate in a non-canonical way: K_v_ 11.1 (hERG1), for example, is strictly associated with the β1 subunit of integrin adhesion receptors in tumors, and stimulates peculiar intracellular signaling pathways that regulate the metastatic process [30]. Besides the plasma membrane, ion channels are present in intracellular organelles of tumor cells, such as mitochondria, where they play a central role in the regulation of either metabolic state or apoptosis. The expression and role of ion channels in cancers has been extensively reviewed by us and other authors. Hence we refer to extensive reviews and related references for further details on this topic [27,28,29,30,31]. The main ion channel types expressed in solid cancers are depicted in Figure 2 and listed in Table 3. A concise picture of the functional aspects of ion channels and a focus on the structural features of voltage-gated ion channels are shown in Box 1 and Figure 3.

Box 1 **Insights into ion channel main structure features.**

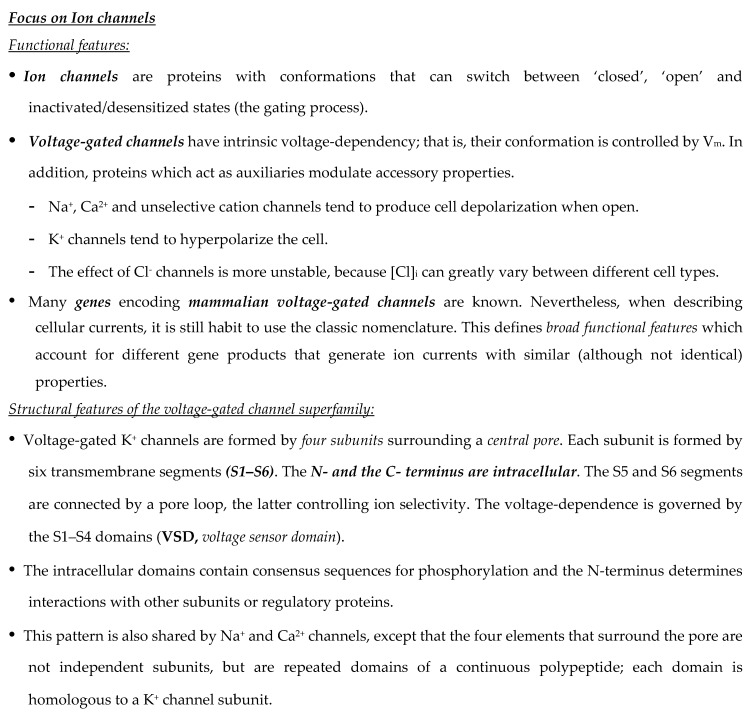

## 5. Development of Antibodies Towards Ion Channels

Ion channels include a very broad collection of structural and functional proteins. Such variety makes their targeting with antibodies a fascinating job. However, the design of antibodies against these structurally complex proteins is often challenging (Figure 2 and Box 1). In particular, the presence of short, poorly accessible extracellular loops (Figure 3) makes the identification of antibodies targeting ion channels from the extracellular side very complex work [111]. Overall, when developing an antibody towards an ion channel, several characteristics of the protein along with the difficulties in protein expression and manipulation, as well as in screening, must be taken into account [112]. Considering the above-mentioned issues, the development of monoclonal antibodies against ion channels still remains a challenge, justifying why only very few antibodies (Table 5) against those ion channels that are expressed in solid tumors have been developed so far [113].

The only example of an antibody targeting a cancer-related ion channel (the purinergic receptor P2X7) which has recently entered into the clinic with the potential to be approved as a first-generation therapy is BIL010t. BIL010t is a polyclonal antibody that targets a conformational epitope of the channel in its non-functional form (nfP2X7, Biosceptre). Given the hurdles faced in the aforementioned development of the antibody, some agonist antibodies against the same antigen were also developed. One of them is capable of inducing the cell death of P2X7-positive T cells, hence offering the possibility of a potential application for onco-immunotherapy [114]. Fully human antibodies targeting the Orai1 protein were raised through the immunization of the “Xenomouse” using U2OS cells overexpressing human Orai1 as immunogens. One of them was able to impair cell proliferation in peripheral blood human T lymphocytes [115,116]. In 2014, a well-conceived and simple strategy to isolate functional antibodies targeting the voltage-dependent Na^+^ channel, Nav1.7, was published [117]. To this purpose, mice immunization was performed using a peptide (VELFLADVEG) located in the loop between the S3 and S4 helices in domain II (Box 1 and Figure 3). Although almost sixteen different antibodies were isolated against TrpA1, all showing high immunogenicity, their therapeutic potential was considered poor due to their lack of potency. A mAb against Eag1 was isolated immunizing mice with a fusion protein composed by residues 374–452 of the E3 loop between the S5–S6 transmembrane segments (i.e., the only scarce extracellular portion which might be exposed and thus targetable by an antibody). The latter was fused to the C-terminal tetramerization domain of the channel (residues 872–932) [118]. The molecule was able to inhibit Eag1 currents in HEK cells transfected with the channel and gave good results for the in vivo imaging of tumor xenografts but lacked biological activities. To induce apoptosis in Eag1-positive tumor cells, an anti-Eag1 scFv derived from the aforementioned mAb was joined to the tumor necrosis factor-related apoptosis-inducing ligand (TRAIL) [119]. This antibody, scFv62-TRAIL, was demonstrated to be a potential tool to overcome resistance to drugs. Overall, such findings demonstrate the possible application of these antibodies in cancer diagnosis as well as for targeted cancer therapy and theranostics [120].

## 6. Ion Channels in Cancer Diagnostics: The Story of K_v_11.1/hERG1

One of the ion channels that is over-expressed and deregulated in human cancers is the voltage-dependent K^+^ channel, K_v_11.1, also known as hERG1. In humans, hERG1 is physiologically expressed only in selected tissues: cardiac myocytes (where it contributes to the repolarizing potassium current I_Kr_), pancreatic beta cells and neuronal cells of some selected areas of the CNS [49]. Since its first discovery in 1995 [86,124], hERG1 has been shown to be aberrantly expressed in human cancers of different histogenesis. In cancer cells, hERG1 modulates the main cancer-related intracellular signaling pathways (FAK, ERK, AKT, NFkB, HIF-α, small GTPases, etc.) and hence drives many characteristics of neoplastic progression. Some examples, related to solid cancers, are reported in Table 5. Overall, many data were obtained both in vitro and in vivo, supporting the notion that hERG1 can be considered a novel cancer biomarker.

### 6.1. Development of Anti-hERG1 Antibodies

While only a few examples of antibodies targeting cancer-related ion channels [113] are detectable in literature so far, our research group has developed a mAb directed against hERG1, which turned out to be applicable for diagnostic purposes through IHC [88,91]. The hERG1-mAb was developed through the immunization of Balb/c mice following the Hybridoma Technology methodology and using a 14-amino acid synthetic peptide which encompasses the extracellular S5-P loop of the protein (highlighted in green in Figure 3). The specific sequence is EQPHMDSRIGWLHN. One out of the positive clones obtained from cell fusion, clone A12, showed the best performances in biological assays and was thus patented (patent Ref. n° FI2006A000008). Thanks to the use of this antibody, strong scientific evidence has been provided demonstrating that hERG1 represents a novel cancer biomarker in patients with both solid cancers and hematologic malignancies [30,125]. A summary of the main clinical evidence obtained so far in solid cancer, especially those arising from the gastrointestinal tract, are detailed below.

### 6.2. Evidence for hERG1 Being a Novel Tumor Biomarker for in Vitro Diagnostics (IVD)

The hERG1-mAb has given encouraging results in different clinical studies, when more than 1500 human tumor samples were analyzed through IHC (see Table 5), reaching a high diagnostic and prognostic value for surgeons and clinical oncologists. The same antibody (and its engineered derivative described in paragraph 6.3) may have another clinical application in the endoscopic setting to detect hERG1 in pre-cancerous or cancerous lesions of GI tracts. In fact, hERG1 is over-expressed in Barrett’s esophagus (BE), a precursor lesion for Esophageal Adenocarcinoma (EA), while absent in normal esophageal mucosa [95] and can identify patients with higher probability to malignant progression towards EA [97]. In other words, the hERG1 biomarker could identify high-risk BE patients and might be exploited for endoscopic surveillance of BE patients, thus allowing an early EA diagnosis.

hERG1 is also highly expressed in primary Gastric Cancer (GC): a study performed on 508 surgical samples showed a hERG1 immunoreactivity in 69% of cases, with a statistically significant negative prognostic impact in early-stage GC and in precancerous lesions (gastric metaplasias and dysplasias) [96]. In particular, hERG1 expression in gastric metaplastic/dysplastic lesions could determine an innovative prognostic marker of progression towards GC of the intestinal histotype.

Much work has been done evaluating hERG1 expression in colorectal cancer (CRC). In the early stages, (TNM stage I and II) CRC hERG1 associates with Glut-1, VEGF-A, CA-IX, and EGFR, and behaves as an independent negative prognostic factor. In metastatic CRC (TNM stage IV), hERG1 represents a factor of positive response to anti-angiogenesis therapy (bevacizumab) [126]. In particular, hERG1-positive patients have a lower risk to progress during bevacizumab treatment. hERG1 can hence be proposed as a prognostic biomarker to identify patients to be treated with antiangiogenic agents, both in first- and second-line treatments.

In Pancreatic Ductal Adenocarcinoma (PDAC), hERG1 is expressed in roughly 60% of surgically resectable (TNM stages II and III) cases. By using our hERG1-mAb and applying a double scoring system, based on both signal intensity and percentage of labeled cells, a high hERG1 scoring was significantly associated with worse prognosis, both in the univariate and multivariate analysis. These results thus indicate hERG1 as an independent prognostic factor of worse prognosis in PDAC [88].

Finally, similar data were obtained by Pointer and colleagues [127], using the same mAb developed by our group. The authors concluded that hERG1 can be considered a potential Glioblastoma Multiforme (GBM) survival marker, since patients whose tumor was positive for hERG1 had a shorter survival compared to hERG1-negative cases. In addition, hERG1 behaved as a positive biomarker of therapy response, since those patients whose tumor was hERG1 positive and were treated with chemotherapy plus a hERG1 blocker (for the treatment of co-morbidities) had a longer survival compared to patients not treated with a hERG1 blocker. This finding led the authors to conclude that already approved hERG1 blockers might be considered as adjuvant therapy in high hERG1-expressing GBM patients [127].

All the above-mentioned results were obtained through IHC, using the anti-hERG1 mAb developed by us. Such a tool was hence very important to propose hERG1 as a potential prognostic marker. The translation potential of such data was corroborated by the possibility of detecting hERG1 in vivo, after its labeling with Alexa-680. In preclinical mouse models, the labeled mAb was able to identify hERG1-expressing PDAC tumors either in PDAC xenografts or in transgenic mice that develop tumor in the pancreas due to the expression of mutated Kras and Trp53 in pancreatic ductal cells [128]. Although, the anti-hERG1 mAb showed valuable proof of concept for in vivo use in preclinical mouse models, the antibody has been extensively implemented and has given promising results as an in vivo imaging tool after its engineering in the scFv format [122] (see below).

### 6.3. Targeting hERG1 for Molecular Imaging

Moving from the monoclonal antibody, we have developed a single-chain variable fragment antibody, anti-hERG1scFv. The antibody was mutagenized, substituting a phenylalanine residue in the third framework of the VH domain with a cysteine residue. The resulting scFv–hERG1–Cys showed much higher stability and protein yield, with better affinity and more advantageous binding kinetics, compared to the parental anti-hERG1scFv. The scFv–hERG1–Cys properly bound the native hERG1 antigen expressed on cells, was stable in serum, and displayed a fast pharmacokinetic profile (half-life of 3.1 h) once injected intravenously in nude mice. Moreover, no general toxicity or cardiac toxic effects were detected. The in vivo distribution of an Alexa Fluor 750 conjugated scFv–hERG1–Cys showed a good tumor-to-organ ratio, ideal for visualizing hERG1-expressing tumor masses in vivo. Such findings allowed us to state that the scFv–hERG1–Cys possesses features which make it a suitable tool for application in cancer molecular imaging ([122], patent Ref: 102017000083637).

The scFv was further developed in order to produce a bispecific antibody in the format of scDb, directed against the hERG1–β1 complex, which is a macromolecular complex formed between hERG1 and β1 integrins which selectively occurs in cancers [102]. Such an antibody, once tested through IHC on both CRC and PDAC paraffin-embedded samples, confirmed its specificity for hERG1/β1 complex. Overall, the scDb–hERG1–β1 antibody could be used as a potential new treatment for cancer patients and as an early molecular diagnostic marker, thus configured as one of the first examples of companion diagnostics targeting ion channels ([123], unpublished data).

## 7. Conclusions and Future Perspectives

Cancer diagnosis has been greatly affected by antibody application. The advent of a completenew class of antibodies, represented by recombinant antibodies with smaller sizes but retained specificities, has increased the possible uses of such a class of proteins for both cancer diagnosis and even for a theranostic approach to cancer. Considering possible novel biomarkers, ion channels are emerging as a new class of proteins and potential novel cancer biomarkers, since they are highly expressed in cancers and are involved in cancer establishment and progression. So far, due to the high complexity of such proteins, only a few antibodies have been developed against ion channels. Hence, there is still a lack of appropriate ion channel mAbs described in the literature and applied either in preclinical or in clinical trials. For these reasons, the example we propose in the present review regarding the antibodies developed against hERG1 by our group will also be remarkably interesting for market opportunities and new targets in the global clinical pipeline.

## 8. Patents

In the present review, we have extensively reviewed the work accomplished using the antibodies patented under the following patent references, n° FI2006A000008, patent Ref: 102017000083637. It is worth noting that the anti-hERG1 antibody developed by the University of Florence was licensed to MCK Therapeutics.

## Figures and Tables

**Table 1:** Antibody-based in vitro diagnostics (IVDs) which are already approved by the FDA (Federal Drug Administration) and/or EMA (European Medicine Agency) and used for cancer diagnosis. CTA, Cancer-testis antigen; CEA, carcinoembryonic antigen; PSMA, prostate-specific membrane antigen; TAG-72, tumor-associated glycoprotein 72; PDL-1, programmed death-ligand 1; HER 2, human epidermal growth factor receptor 2; EGFR, epidermal growth factor receptor; ALK, anaplastic lymphoma kinase.

IVD Commercial Name	Manufacturer	Antigen	Antibody Format	Tumor Type	Diagnostic Significance	Possibility of Companion Diagnostic
Humaspect^®^	Organon Teknica	CTA	Humanized mAb	Colorectal cancer/tumor detection		NA
CEA-scan^®^	Immunomedics	CEA	Murine Fab fragment	Colorectal cancer/tumor detection	Tumor marker, Prognostic marker	NA
ProstaScint^®^	Cytogen	PSMA	Murine mAb	Prostate adenocarcinoma/tumor detection	Prognostic marker	NA
Verluma^®^ (Diagnostic)	Boehringer Ingelheim, NeoRx	CD-20	Murine Fab fragment	Small-cell lung cancer/tumor detection		NA
OncoScint^®^	Cytogen	TAG-72	Murine mAb	Colorectal and ovarian cancer/tumor detection		NA
PD-L1 IHC 22C3 pharmDx	Dako North America Inc.	PDL-1	Murine mAb	Non-small-cell lung cancer/tumor detection		Yes Keytruda (pembrolizuma)—BLA 125514
VENTANA PD-L1(SP142) Assay	Ventana Medical Systems, Inc.	PDL-1	Rabbit mAb	Non-small-cell lung cancer and urothelial cancer/tumor detection		Yes Tecentriq (atezolizumab)—NDA 761034/S012
Dako EGFR pharmDx Kit	Dako North America, Inc.	EGFR	Murine mAb, (clone 2-18C9)	Colorectal cancer/tumor detection		Yes Erbitux (cetuximab)—BLA 125084 Vectibix (panitumuma)—BLA 125147
PATHWAY anti-Her2/neu (4B5)	Ventana Medical Systems, Inc.	HER2	Rabbit mAb	Breast cancer/tumor detection		Yes Herceptin (trastuzumab)—BLA 103792
Bond Oracle HER2 IHC System	Laica Biosystem	HER2	mAb (CB11 clone)	Breast cancer detection	Tumor marker, Prognostic marker	Yes Herceptin (trastuzumab)—BLA 103792
HercepTest	Dako Denmark A/S	HER2	Rabbit mAb	Breast cancer detection	Tumor marker, Prognostic marker	Yes Herceptin (trastuzumab)—BLA 103792
VENTANA ALK (D5F3) CDx Assay	Ventana Medical Systems, Inc.	ALK	Rabbit mAb	Non-small-cell lung carcinoma detection	Tumor marker, Prognostic marker	Yes Zykadia (ceritinib)—NDA 205755 Xalkori (crizotinib)— NDA 202570 Alecensa (alectinib)—NDA 208434

**Table 2:** Antibodies for in vivo use in tumor imaging diagnostics approved by the FDA and/or EMA and present on the market. CEA, carcinoembryonic antigen; Tc, technetium; In, indium.

**A) Monoclonal Antibodies**					
**Commercial Name**	**Company**	**Antibody Format**	**Antigen**	**Conjugated Probe**	**Tumor Application**
					
Capromab pendetide (ProstaScint)	Cytogen	7E11-C5.3, mouse IgG1	100-kDa glycoprotein	^111^In	Prostate carcinoma
Votumumab (HumaSPECT)	Intracel	88BV59, human IgG3	Altered cytokeratins	“	Colorectal, ovarian and breast carcinoma
Ibritumomab tiuxetan (Zevalin)	Spectrum Pharms	2B8, mouse IgG1	CD20	“	Non-Hodgkin lymphoma
Tositumomab (Bexxar)	SmithKline Beecham	B1, mouse IgG2a	“	“	“
Cetuximab	Cetuximab-IRDye800CW	Human-murine chimeric monoclonal antibody (mAb)	EGFR	IRDye800CW	Head and neck squamous cell carcinoma, pancreatic cancer
Trastuzumab	(^111^In-DTPA)n-trastuzumab-(IRDye 800CW)	Humanized monoclonal antibody	HER2	“	Breast cancer
**B) Engineered Antibody Fragments**					
Arcitumomab (CEA-Scan)	Immunomedics	IMMU-4, mouse IgG1 Fab’	CEA	^99m^Tc	Colorectal and ovarian carcinoma
Nofetumomab merpentan (Verluma)	Boehringer Ingelheim	NR-LU-10, mouse IgG2b Fab	40-kDa glycoprotein	“	Small-cell and non-small-cell lung carcinoma
Bectumomab (LymphoScan)	Immunomedics	LL2, mouse IgG2a Fab’	CD22	^99m^Tc	“
Igovomab (Indimacis-125)	CIS Bio International	OC125, mouse IgG1 F(ab’)2	CA125	^111^In	Ovarian cancer

**Table 3:** Main ion channels expressed in solid cancers, with their role in tumor biology. Ion channels are indicated using the HGNC (HUGO Gene Nomenclature Committee) classification. Cancers are indicated using the acronyms as follows: BC, breast cancer; PDAC, pancreatic cancer; CRC, colorectal cancer; LC, lung cancer; HC, head cancer; PC, prostate cancer; EC, esophageal cancer; GC, gastric cancer. Firstly, voltage-gated potassium channels are reported, followed by calcium-activated potassium channels. For each channel, it has been indicated whether it is an early biomarker (eb), which allows early detection of the cancer in a noninvasive way and thus the secondary prevention of the cancer; a prognostic biomarker (pb), which is a clinical or biological characteristic that provides information on the likely course of the disease and gives information about the outcome of the patient; or a tumor marker (tm), which are proteins that can be elevated by the presence of one or more types of cancer.

Name	Tumor Type	Role in Tumor Biology	Exploitation for Diagnostic Purposes	Reference
***Potassium***				
KCNH1	BC, EC, PDAC, CRC	Modulation of cell cycle and proliferation	tm, pm	[32]
KCNH2		Reviwed in detail in Table 4		
KCNA3	PC, PDAC, CRC	Tumor progression, Metastatic spreading	tm, pm	[33,34,35]
KCNA5	“	“	tm	[36]
KCNQ1	LC	Hypoxia Resistance	tm	[37]
KCNQ5	CRC	Cell proliferation	tm	[38]
KCNMA1	BC, PC	Modulation of cell cycle and proliferation, Cell proliferation	tm, pm	[39,40]
KCNN4	BC, PDAC	Modulation of cell cycle and Cell proliferation	tm, pm	[41,42]
KCNC4	CRC	“	tm	[43]
KCNJ3	BC, PDAC	Modulation of cell cycle and Cell proliferation	tm, pm	[43,44]
KCNK5	BC	Modulation of cell cycle and proliferation	tm, pm	[45]
KCNK9	BC, CRC	“	pm	[46,47]
***Sodium***				
SCN5A	BC, CRC	Cell proliferation and invasiveness	tm	[48,49]
SCN9A	PC, LC	Migration and metastatic spreading	tm, pm	[50,51]
***Calcium***				
CACNA2D	BC	Cell proliferation	NA	[52]
CACNA1H	PC	“	tm	[53]
CACNA	EC, CRC	Cell proliferation, Cell invasion	tm	[54,55]
CACNA2D3	GC, HC	Tumor suppression	tm	[56,57]
ATP2C1	BC	Cell proliferation	tm, pm	[58]
ATP2B2	“	“	tm	[59]
ORAI1	BC, PC	Cell invasion, Cell survival	tm	“
ORAI3	BC, LC	Cell proliferation and invasiveness	tm	[60]
***Chloride***				
ANO1	BC, PDAC	Cell proliferation and invasiveness	tm	[61,62]
CLCA1	CRC	Cell proliferation and invasiveness	tm	[63]
CLCA2	BC	Tumor suppression	tm	[64]
	CRC	Cell differentiation	tm	[65]
CLCA4	“	Tumor suppression	tm	[65]
CLIC1	CRC, GC	Migration and metastatic spreading, Cell proliferation, apoptosis, invasiveness	tm	[66]
CLIC3	PDAC	Cell survival	tm	[67]
***TRP***				
TRPM8	BC, PC, PDAC	“	tm, pm	[68,69,70]
TRPM7	BC, PDAC	Cell proliferation and invasiveness	tm	[71,72]
TRPA1	LC	Cell survival	tm, pm	[73]
TRPC1	BC, PC, LC	Cell proliferation, Migration and metastatic spreading AND Cell survival	tm	[74,75]
TRPC3	BC, LC	Cell proliferation, Cell survival	tm	[76]
TRPC4	LC	Cell proliferation, Cell survival	tm	[77]
TRPC6	LC, EC	“	tm, eb, pm	[78,79]
TRPV1	PDAC	Cell proliferation	tm, pm	[80]
TRPV4	BC	Migration and metastatic spreading	tm	[81]
TRPV6	PC	Reduction of cell growth	tm	[82]

**Table 4:** Different human solid tumors in which the Kv11.1 (hERG1) ion channel is expressed, enlightening pre-clinical and clinical aspects in which it is involved. The different types of cancers have been indicated using acronyms as follows: Head&Neck, HNSCC; Oral squamous cell carcinoma, OSCC; Glioblastoma Multiforme, GBM; NB, neuroblastoma; BC, breast cancer; PDAC, pancreatic cancer; P. NET, pancreatic neuroendocrine tumor; CRC, colorectal cancer; LC, lung cancer; HC, head cancer; PC, prostate cancer; EC, esophageal cancer; GC, gastric cancer; BE, Barrett’s Esophagus; EC, endometrial cancer; OC, ovarian cancer; ML, melanoma; OSR, osteosarcoma.

Tumor Type	hERG1 Involvement in Cancer Biology Aspects			References
	**Effect in vitro**	**Signaling pathway affected**	**Consequences of hERG1 blockade in vivo**	
HNSCC OSCC	HNSCC: Migration OSCC: Invasiveness	Sphingosine 1-phosphate (S1P) receptors	NA	[83,84]
GBM	Proliferation, Ki67	Vegf	NA	[85]
NB	Cell cycle regulation	NA	Reduction of mean tumor weight in mice treated with hERG1 and hERG1b inhibitor ZC88	[86]
LC	Proliferation (small-cell lung cancer (SCLC))	NA	NA	[87]
PDAC	Proliferation Migration Invasiveness	EGF-R signaling pathway	Block of local growth and of metastatic spread	[88,89,90]
CRC	Invasiveness Angiogenesis Metastasis	Akt, NFkB, HIF-1/2α, VEGFHIF-1/2α	Block of local growth and of metastatic spread	[91,92,93,94]
BE, EC	NA	NA	NA	[95,96]
GC	Cell proliferation Apoptosis VEGF-A secretion	AKT, pAKT, HIF2α, VEGF	Block of local growth Combined activity of hERG1 blockers and anti-VEGF-A antibodies (Bevacizumab)	[97,98,99,100]
P. NET	NA	NA	NA	[101]
BC	Induction of cell senescence Activation of p21/waf transcription Metastasis	Ras-dependent DNA damage Actin assembly	Block of metastatic spread	[102,103]
EC	NA	NA	NA	[104]
OC	Proliferation	NA	NA	[105,106,107]
ML	Proliferation Migration	MAP kinase/c-fos pathway.	NA	[108,109]
OSC	Proliferation, Migration, Apoptosis	PI3K/Akt/NFkB	NA	[110]

**Table 5:** Ion channel-targeting antibody-based tools developed or under development. P2X7, ionotropic ATP-gated receptors; Eag-1, ether-à-go-go-1.

Target	Ion Channel Type	Antibody Format	Assay	Reference
P2X7	Ligand-gated	mAb	Cell-binding assays, whole-cell patch clamp and recognition of native P2X7	[114]
Orai1	Calcium release-activated channel	mAb	Cell-binding assays, store-operated calcium influx, and NFAT-dependent luciferase activity	[115]
Orai1	Calcium release-activated channel	mAb peptide based	ELISA cell-binding assays, calcium flux, Orai1 internalization, and T-cell proliferation	[116]
TrpA1	Transient receptor potential channel	mAb	Cell-binding and radioactive calcium uptake assay	[73]
Nav1.7	Voltage-gated	mAb	ELISA using purified sensor domain protein and whole-cell patch clamp	[117]
Eag-1	Voltage-gated	mAb	ELISA and SPR, whole-cell patch clamp	[119]
hERG1	Voltage-gated K^+^ channel	mAb	ELISA and SPR, whole-cell patch clamp and IHC	[121]
	“	scFv	ELISA and SPR, IHC ex vivo, in vivo imaging	[122]
hERG1/β1	Voltage-gated K^+^ channel	scDb	In vivo tumor targeting	[123]
